# Pollen proteomics: from stress physiology to developmental priming

**DOI:** 10.1007/s00497-016-0283-9

**Published:** 2016-06-08

**Authors:** Palak Chaturvedi, Arindam Ghatak, Wolfram Weckwerth

**Affiliations:** Department of Ecogenomics and Systems Biology, Faculty of Sciences, University of Vienna, Althanstrasse 14, 1090 Vienna, Austria; School of Biotechnology and Bioinformatics, D.Y. Patil University, Sector No-15, CBD, Belapur, Navi Mumbai, India; Vienna Metabolomics Center (VIME), University of Vienna, Vienna, Austria

**Keywords:** Pollen, Pollen development, Proteomics, Heat stress, Developmental priming, Defense priming

## Abstract

*****Key message***:**

**Pollen development and stress.**

**Abstract:**

In angiosperms, pollen or pollen grain (male gametophyte) is a highly reduced two- or three-cell structure which plays a decisive role in plant reproduction. Male gametophyte development takes place in anther locules where diploid sporophytic cells undergo meiotic division followed by two consecutive mitotic processes. A desiccated and metabolically quiescent form of mature pollen is released from the anther which lands on the stigma. Pollen tube growth takes place followed by double fertilization. Apart from its importance in sexual reproduction, pollen is also an interesting model system which integrates fundamental cellular processes like cell division, differentiation, fate determination, polar establishment, cell to cell recognition and communication. Recently, pollen functionality has been studied by multidisciplinary approaches which also include OMICS analyses like transcriptomics, proteomics and metabolomics. Here, we review recent advances in proteomics of pollen development and propose the process of developmental priming playing a key role to guard highly sensitive developmental processes.

## Introduction

Plants are always in an intimate contact with the environment and continuously challenged by unfavorable conditions which include biotic and abiotic stresses like lack/excess of water, light, nutrients, high/low temperatures and many more. Environmental fluctuations have a major influence on plant performance, especially during the reproductive cycle, and can reduce crop productivity (Levitt 1980; Hirt and Shinozaki 2004; Barnabas et al. 2008; Bokszczanin et al. 2013). In order to cope with these environmental constraints, most of the plants develop defense mechanisms which include changes in gene expression, resulting in changes in protein translation and metabolic reprogramming. These mechanisms lead to metabolic adaptation and plant survival under stress condition (Conrath 2011; Krasensky and Jonak 2012; Tanou et al. 2012). Currently, we have only a limited understanding of these highly complex short and long-term acclimation and adaptation processes. At the same time, novel biochemical and bioanalytical tools such as genome sequencing, transcriptomics, proteomics, metabolomics, genome-scale metabolic reconstruction and lipidomics enable more in-depth analyses of these processes than ever before (Weckwerth 2011; Astarita and Ollero 2015). Therefore for the better sustainability of crops, it is necessary to explore and understand the genetic and molecular background of systemic stress response mechanisms along with physiological parameters.

**Fig. 1:**
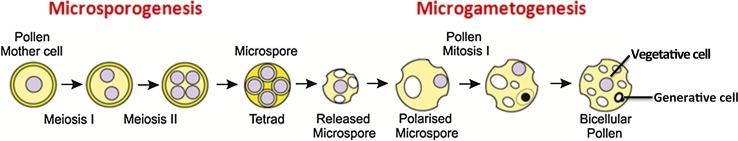
Schematic diagram representing different developmental stages of pollen. The reproductive system consists of two phases, i.e., microsporogenesis and microgametogenesis [figure adaptation from McChormick (1993), Giorno et al. (2010)]

**Fig. 2:**
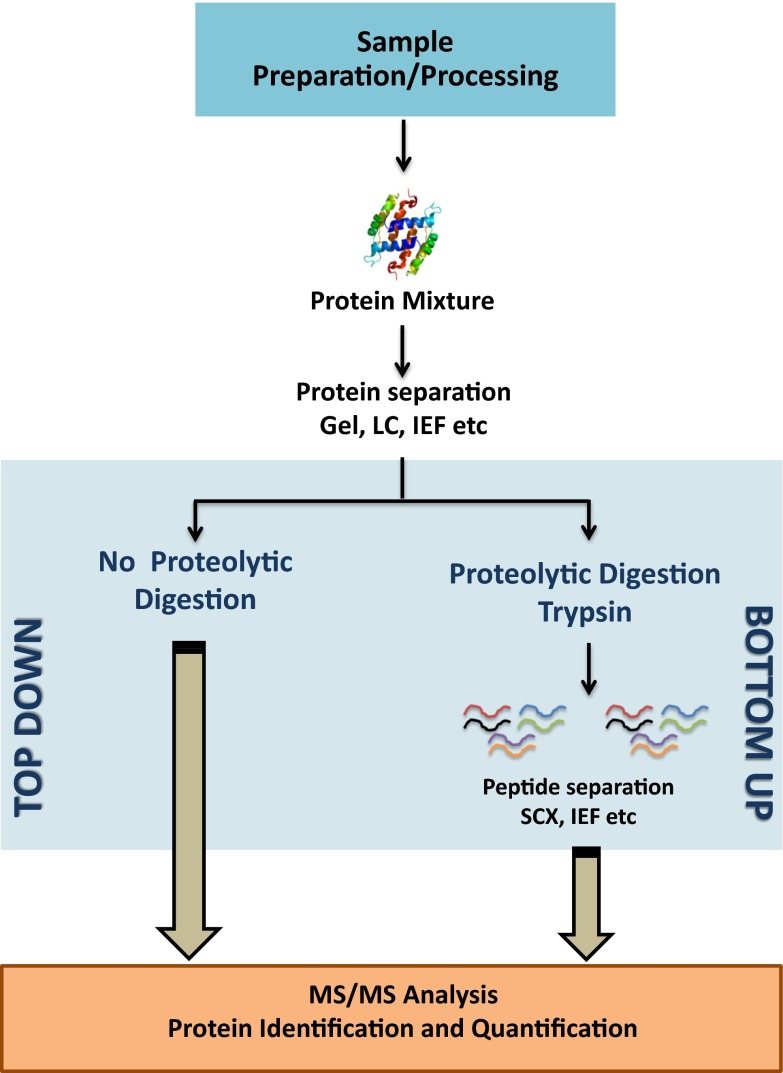
Schematic representation determining comparison between different proteomic strategies employed to protein mixture, i.e., *top-down* versus *bottom-up*

The most important process for plant productivity is the generative life cycle of a plant, especially with a focus on crop plants. The life cycle of angiosperm oscillates between diploid (sporophyte) and haploid (gametophyte) generations (McChormick 1993). In order to reproduce, male gametophyte or pollen grain plays a very important role in the flowering plants. The life cycle of male gametophyte development is divided in two major phases: (1) pollen development [which leads to the formation of mature pollen (bicellular/tricellular) by two sequential processes known as microsporogenesis and microgametogenesis] (Fig. 1) and (2) the tuberization process (begins when desiccated mature pollen falls on to the stigma, continues with pollen tube growth which leads to the double fertilization). Pollen development is initiated in the anthers by microsporocytes which undergo meiosis, thereby forming tetrads of haploid microspores. This stage is completed when specific microspores with one central haploid nucleus are released from tetrads with the help of various enzymes secreted by the tapetum (Bedinger 1992). Subsequently, each released microspore develops a large vacuole which leads to the migration of the microspore nucleus to the periphery near the cell wall (Owen and Makaroff 1995; Yamamoto et al. 2003). The microspore then undergoes a first mitosis [pollen mitosis I (PM I)] giving rise to a large vegetative cell and smaller generative cell (asymmetric mitosis). Finally, a second division of the generative cell [pollen mitosis II (PM II)] completes the formation of the male gametophyte and leads to the formation of two sperm cells (male gametes). The two sperm cells form the male germ unit (MGU) which is delivered to the embryo sac where double fertilization takes place. Some plant species have tricellular pollen grain, for example, *Arabidopsis thaliana*, where PM II takes place prior anthesis, whereas in bicellular pollen (e.g., *Lilium longiflorum*), PM II takes place after germination of the pollen tube (Borg et al. 2009).

During dehiscence, dehydration of pollen takes place which reduces the water content down to 40–58 % (Barnabas 1985). Further, the desiccated pollen is released from the anther. Finally, in contact with the stigma, pollen rehydrates and grows a pollen tube which delivers the two sperm cells to the ovule where double fertilization takes place (Boavida et al. 2005). Any disturbance, e.g., high-/low-temperature fluctuations, in this developmental process can lead to male infertility of a plant (Sakata and Higashitani 2008; Wassmann et al. 2009). Due to the elementary function of sexual reproduction, pollen development integrates fundamental cellular processes like cell division, differentiation, fate determination, polar establishment, cell to cell recognition and communication (Procissi et al. 2001; Dai et al. 2007; Borg et al. 2009).

In mature pollen the vegetative cell is a reservoir for carbohydrates and lipids along with transcripts and proteins which play a primary role for the rapid development of pollen tube (Pacini 1996). Osmoprotectants (like disaccharides, proline, glycine, betaine) which play a protective role for proteins and membranes during dehydration are also stored in mature pollen (Schwacke et al. 1999). It is also assumed that some mRNAs are presynthesized and stored in mature pollen. Later, they are translated into proteins during the germination process (Mascarenhas 1989, 1990).

During pollen development, the tapetum plays a very crucial role and performs a variety of important functions: (1) providing nutrients to the microspores (regarded as nurse cells in the mammalian system), (2) release of haploid microspores from the enclosing callose wall of the meiotic tetrad by secretion of the β-1, 2-glucanase or callase (Pacini et al. 1985). The secretion of callase is very important for the normal development of pollen. In many studies it is reported that slight modification in the secretion of callase or callase gene expression can lead to destruction of developing microspore or complete/partial male sterility (Izhar and Frankel 1971; Worrall et al. 1992). (3) Tapetal cells produce precursors for biosynthesis of the outer pollen wall or exine which include deposition of cell fragments on the surface of mature pollen known as typhrine or pollen kit. The main function is to protect pollen grain from dehydration and to attract and adhere insect pollinators (Bedinger 1992).

Genetic and cytological studies reveal that sporophytic mutations also affect tapetum cells which cause male sterility (Beadle 1932; Albertsen and Phillips 1981). Hence, isolation of the sporophytic male sterility genes can provide us the specific function of the tapetum. Therefore, to control and completely understand the process of fertility in the flowering plants, tapetal cells are an excellent model for the application of genetic engineering tools. Mariani et al. (1990) induced male sterility in tobacco via selective destruction of the tapetum by fusing a promoter of a specific gene expressed in tapetal cells to a cytotoxic ribonuclease gene.

Proteins are key biomolecules in the living organism. In contrast to genes they are actively involved in metabolism, development, reproduction, defense mechanisms and many further processes which define a living system. The term proteomics was defined in 1996 by merging two words from “***prot***ein” and “gen***omics***” (Wilkins et al. 1996; James 1997). It can be defined as “the efficient and/or standardized analysis of all the proteins present in tissues, cells or the subcellular compartment.” Proteomics is per se an untargeted technology aiming for the analysis of the complete proteome of the organism of interest but can also include targeted identification and quantification of specific proteins/peptides. Proteomic profiling determines the abundance level of the proteins in different cell types and tissues at any given state as well as in between the samples of various combinations. Using enrichment techniques for phosphoproteomics in vivo posttranslational protein modifications (PTMs) can be detected in pollen (Fíla and Honys 2012; Chen et al. 2010; Silva-Sanchez et al. 2015; Fila et al. 2016). Furthermore, functional protein–protein interactions make proteomics even more complex and challenging as sequencing a genome (Zhang et al. 2013).

Experimental strategies employed in the proteomics approach (i.e., top-down vs. bottom-up) are illustrated by the schematic representation (Fig. 2). Proteomic analysis should comprise the following strategies and analyses: (1) separation, identification and quantification of proteins based on 2D gel electrophoresis or gel-free shotgun proteomics using liquid chromatography tandem mass spectrometry (LC–MS/MS) platforms; (2) annotation of protein functions and protein functional networks through protein mapping, characterization of PTMs and protein–protein interactions; (3) bioinformatic analysis and the use of databases of the model and non-model plant species (Holman et al. 2013).

Recently, gel-free protein separation and second generation of proteomic techniques like multidimensional protein identification technology (MudPIT), quantitative proteomic approaches such as isotope-code affinity tags (ICATs), targeted mass tags (TMTs), isobaric tags for relative and absolute quantitation (iTRAQ), label-free shotgun proteomics using mass accuracy precursor alignments (MAPA) or metabolic labeling techniques have been widely used for descriptive and comparative proteomic studies (Matros et al. 2011; Hoehenwarter et al. 2008, 2011; Chen et al. 2010; Egelhofer et al. 2013; Chaturvedi et al. 2015).

Plants are multicellular organisms which have different cell types. Each cell type has a specific function in development and growth of the plant. Application of proteomics in plant physiology was mainly focused on organs and tissues which contain mixtures of different cell types. However, this decreases the resolution and selectivity to understand the role and function of a specific protein in a specific cell type (Dai and Chen 2012). In recent years, more cell-specific and subcellular proteomic studies emerged (Wienkoop et al. 2004; Glinski and Weckwerth 2006). The majority of the proteomics data for single cell type research is available on bacteria, yeast, cultured cell lines, blood tissues, etc. (Ideker et al. 2001; Diks and Peppelenbosch 2004; Ishii et al. 2007; Pasini et al. 2008). In recent years, more single cell type proteomics research is emerging in plant biology research, but there is always a limitation attributed to the experimental challenges which include harvesting of the individual cells in quantity and quality from the plant tissue. However, dedifferentiated plant cell cultures have clear advantage as they contain genetic information and they are not limited in terms of quantity (Dai and Chen 2012). Studies on cell suspension culture of *Arabidopsis* vacuoles lead to the identification of 1107 proteins (Jaquinod et al. 2007). Similarly, 1528 proteins were identified in rice leaf and seed callus suspension culture (*Oryza sativa*) (Jung et al. 2008), 360 proteins were identified in tobacco (*Nicotiana tabacum*) plastid cell culture (Baginsky et al. 2004), 1367 proteins of 1661 identified protein spots were determined from the suspension culture of *Medicago* (Lei et al. 2005) and 724 proteins were identified in secretome of chickpea (*Cicer arietinum*) (Gupta et al. 2011).

In order to understand the cellular events, function, molecular network of the specific differentiated plant cells and their role in the plant growth and development, proteomics studies are also extended to plant reproductive cells (pollen grain and egg cells) (Holmes-Davis et al. 2005; Noir et al. 2005; Sheoran et al. 2007; Grobei et al. 2009; Zou et al. 2009; Okamoto et al. 2004; Fernando 2005; Dai et al. 2006, 2007; Pertl et al. 2009; Han et al. 2010; Fíla et al. 2012; Fila et al. 2012), leaf epidermal cells, i.e., guard cells and trichomes (Wienkoop et al. 2004; Zhao et al. 2008, 2010), root hair cells (Wan et al. 2005; Brechenmacher et al. 2009; Nestler et al. 2011), mesophyll cells (Zhu et al. 2009), etc. Recently, a study on the membrane proteome of mature pollen was performed with two tomato cultivars (cv. Moneymaker and cv. Red setter) in order to understand the role and function of specific membrane proteins in the development of male gametophyte (Paul et al. 2015). The study revealed a high proportion of membrane-associated proteins involved in energy metabolism such as glycolysis and TCA cycle. The hypothesis is that those proteins are helping during pollen germination and rapid pollen tube growth. Proteomic analysis of embryogenic callus dissected the processes of cellular dedifferentiation and callus formation in lotus using a label-free shotgun proteomics approach (Liu et al. 2015). Protein samples were prefractionated using SDS gel, and each lane of the gels was divided into four fractions. Gel pieces were destained, equilibrated, digested with trypsin as described by Valledor and Weckwerth (2012). Afterwards, tryptic peptides were analyzed using nano-HPLC coupled to LTQ-Orbitrap-MS according to previously published reports (Chaturvedi et al. 2013). The newly annotated genome database of lotus (http://lotus-db.wbgcas.cn) was employed to identify proteins (Ming et al. 2013). In this study, 91 differentially expressed proteins were identified of which 50 % of the proteins were involved in different metabolic activity, 14 and 13 % were binned in the functionality of stress/redox and cell wall, respectively (Liu et al. 2015).

Over the past decades, major advances in genomic analysis have taken place including complete sequence annotation of *Arabidopsis thaliana*, rice (*Oryza sativa*), tomato (*Solanum lycopersicum*) and many other plant genomes (Weckwerth 2011). Proteomics data are also used for functional analysis of those newly sequenced genomes, an approach called proteogenomics (May et al. 2008; Valledor et al. 2012; Weckwerth 2011).

The availability of these comprehensive public sequence databases had a strong impact on proteome and transcriptome research, which in turn significantly helped to understand male gametophyte development at the molecular level (Grobei et al. 2009; Honys and Twell 2004). These studies are associated with transcriptomic profiling of four developmental stages in the *Arabidopsis thaliana* ecotype *Landberg erecta* (uninucleated microspores, bicellular pollen, mature tricellular pollen) and on one stage of the ecotype *Columbia* (mature pollen) (Honys and Twell 2003; Zimmermann et al. 2004; Pina et al. 2005). Considering all these datasets, ~5000–7000 genes were shown to be expressed in the mature male gametophyte and ~14,000 genes were shown to be expressed in all pollen developmental stages (Honys and Twell 2003). Similar, transcriptomic analyses were performed with *Brassica napus* and rice pollen which uncovered strong variation at the transcriptome level from the microspore to the mature pollen stage. So far, the transcriptome of early stages such as microsporocytes, meiosis and tetrads have not yet been studied extensively due to limited access to sufficient sampling material (Wei et al. 2010; Whittle et al. 2010).

## Proteomic studies on pollen development

The first proteomic analysis on early pollen development was performed using rice anthers (young microspore stages) as a material (Imin et al. 2001). In this study, auricle distance (AD) was correlated with developmental stage of the rice microspore (due to the limitation that tetrad and early microspore stages were not separated into two different stages, they are termed together as “young microspore stage”). In total, 4000 anther protein spots were separated using silver-stained 2D gels, of which 75 spots representing 62 proteins were identified using MALDI-TOF MS. Kerim et al. 2003 generated proteome maps from six developmental stages of anther (i.e., anther material correlated/represented six pollen developmental stages). In this analysis, it was observed that 150 proteins spots were consistently changed in the course of development and only 40 spots representing 33 proteins were uniquely identified. The main functions of the identified proteins included carbohydrate metabolism, cell wall and cytoskeleton. Proteins associated with sugar metabolism, cell elongation and cell expansion (like fructokinase, β-expasin and profilin) were also identified and upregulated. More studies related to proteomic analysis were focused mainly on mature pollen and in vitro grown pollen tubes due to an easy availability of the material; such analyses include *Arabidopsis*, lily (*Lilium longiflorum*), tomato (*Lycopersicon esculentum*), rice (*Oryza sativa*), *Quercus*, pine trees, tobacco (*Nicotiana tabacum*) and others. Proteomic analysis of the Arabidopsis mature pollen led to the identification of 135 unique proteins which were involved in energy metabolism, cell wall metabolism, cell structure and protein synthesis (Holmes-Davis et al. 2005; Noir et al. 2005). Pollen-specific proteins included glycosyl hydrolases, germin-like protein, pectin methylesterase inhibitor, actin-depolymerizing factor, and others. Approximately nine proteins were determined whose corresponding genes were not identified at transcript level (Holmes-Davis et al. 2005; Noir et al. 2005). Functionality of the proteins showed similarity with the rice mature pollen grain (MPGs) proteome (Dai et al. 2006). Many novel proteins were identified in rice MPGs, comprising signaling proteins like protein kinases, receptor kinase-interacting proteins, GDP dissociation inhibitors, C2 domain proteins and cyclophilins. Prohibitin, mitochondrial processing peptidase, ubiquitin fusion degradation protein, AAA1 ATPase represented protein metabolism, followed by glycosylated polypeptides, cellulase synthase like OsCsLf7 involved in cell wall remodeling (Dai et al. 2006).

Recently, studies performed by Chaturvedi et al. (2013) and Ischebeck et al. (2014) have established comprehensive proteome maps of pollen development including two species of the Solanaceae family: tomato and tobacco. To reveal a complete quantitative proteome map, it is important to address also the very early stages: diploid microsporocytes (A), tetrads (B), microspores (C), polarized microspores (D), bipolar mature pollen (E), and additionally desiccated pollen (G), and pollen tubes (H). In these studies a new protocol for the isolation of the early stages was developed and proteins were extracted and analyzed by means of a gel LC–MS fractionation protocol. In total about 3690 unique proteins in tomato (1821 proteins) and tobacco (1869 proteins) were identified and quantified (Chaturvedi et al. 2013; Ischebeck et al. 2014). All the spectra of the identified proteins and their metainformation are stored in the public plant proteomics databases PROMEX (http://promex.pph.univie.ac.at/promex/; Hummel et al. 2007; Wienkoop et al. 2012) with the MetaID: “Lyc escu001” for tomato and Nic taba002 for tobacco pollen dataset, additionally tobacco proteomic dataset is also deposited in the ProteomeXchange (data identifier PXD000469). Quantitative analysis was performed based on ion intensity and peptide count. Remarkable stage-specific protein regulation was observed during the course of development from sporophytic to the gametophytic phase. A proteome map with highly specialized functionality of different stages revealed changes in the metabolic activity. Further high levels of heat-shock proteins were also identified in the very early stages of development. This study revealed a process in which the cells of a specific stage are prepared for the effective progression of development and potentially “primed” for sudden environmental stress (Chaturvedi et al. 2013). A cluster analysis of stage-specific proteins during pollen development was performed. Each stage showed a specific set of proteins thereby revealing stage-specific protein candidates; e.g., in pollen mother cells (microsporocytes) histones (H4, H2A, H3 and H4) were detected which are important proteins of the chromatin structure and thus candidates for the transcriptional regulation. Several heat-shock proteins (HSP 20, HSP 22 and HSP 70) were detected with high levels which might not only protect pollen mother cells under heat stress condition but also prepare the cells to undergo meiotic and mitotic divisions during the next step in the developmental process. Further proteins involved in the cell wall degradation were shown to have increased levels in tetrad stage including β-d-glucosidase which contributes to release the individual microspores. Other protein candidates involved in secondary metabolism, cell division and hormone metabolism showed increased levels in tetrad and polarized microspore stages. These two phases are important for correct cellular development of the male gametophyte. Protein candidates like prohibitin, annexin and subunits of the proteasome were also identified. Further, in mature pollen, proteins that are involved in energy metabolism and cell wall metabolism showed increased levels including malic enzyme, phosphoenolpyruvate carboxylase, cellulase synthase family proteins and others, indicating that mature pollen stores many proteins that are required for rapid growth of the pollen tube. Accordingly, each stage showed a specific reprogramming of the proteome. Because it was observed that many proteins which are typically involved in stress responses (e.g., HSP 70, HSP 22, HSP 20) were upregulated during the ordinary unstressed development, these specific responses were termed as developmental priming (Chaturvedi et al. 2013). Although developmental priming seems to be a protecting mechanism against sudden stresses, it is still unknown whether changes in these proteins have also a developmental function in unstressed plants and might influence pollen viability and the fertilization process.

Based on the analysis, it was hypothesized that specific classes of proteins are synthesized in advance for metabolic demands or environmental stresses to prepare the cells for their specific developmental program or provide stress protection (Chaturvedi et al. 2013; Ischebeck et al. 2014) (see below “Developmental Priming”).

Studies on pollen proteomics provided us an extended knowledge on pollen–pistil interaction and signaling, and many proteins related to pollen allergens were also identified which helped to understand human pollen allergen response (Puc 2003; Mohapatra et al. 2008). Many plant species (e.g., grasses) pollen contains allergens which are majorly water soluble proteins or glycoproteins. These proteins evoke IgE antibody-mediated allergic reactions (Taketomi et al. 2006). Examples are expansin, profilin, group 3 pollen allergens, Ory s1, UGP and extension-like allergen (Dai et al. 2006; Cosgrove et al. 1997). Further studies from Bermuda grass (*Cynodon dactylon)* (Kao et al. 2005), maize (*Zea mays*) and other plants (Corti et al. 2005) which combined proteomics and immunoblotting techniques lead to the identification of novel pollen allergens which include enolase, aldolase, elongation factor 2, pathogenesis-related protein and malate dehydrogenase. Proteomics analysis of the *Lilium davidii* pollen and pollen tube revealed that the clathrin-dependent endocytosis pathway plays a crucial role in polarity and tip growth (Han et al. 2010). Several plasma membrane-related proteins were also identified (calcium-dependent kinase, mitogen-active protein kinase 7 (MAPK 7), transforming growth receptor interacting protein and gamma adaptin/clathrin assembly protein), and these proteins were not reported previously.

Protein isoforms which are generated during the transcription or posttranslational modification (PTM) processes also play a very important role in pollen development. Very recently a study by Zhu et al. (2014) demonstrated the specific expression of annexin 5 (ann 5) (an isoform of annexin) in mature pollen, suggesting its vital role in *Arabidopsis* pollen development. Similarly, multiple isoforms of proteins having putative role in cell wall metabolism, cytoskeleton dynamics and carbohydrate metabolism showed abundant levels which clearly determined that the posttranslational modification of the proteins plays a crucial role in pollen development. Mature pollen of Arabidopsis and rice also has 23–30 % of proteins with multiple isoforms (Holmes-Davis et al. 2005; Noir et al. 2005; Dai et al. 2006). Fila et al. used enrichment techniques for the analysis of phosphoproteins in response to in vitro activation of quiescent dehydrated pollen (Fila et al. 2012, 2016). Table 1 provides the brief summary of the publications on pollen proteomics.

**Table 1:** Summary of the publications on pollen proteomics

Species	Sample	References
*Arabidopsis thaliana*	Mature pollen	Holmes-Davis et al. (2005)
	Mature and germinated pollen	Zou et al. (2009)
	Mature pollen	Grobei et al. (2009)
	Mature pollen	Noir et al. (2005)
	Mature pollen (phosphoproteomic analysis)	Mayank et al. (2012)
*Lycopersicon esculentum*	Mature pollen	Sheoran et al. (2007)
*Solanum lycopersicum (cv. Red Setter)*	Pollen developmental stages (i.e., pollen mother cell, tetrad, microspore, polarized microspore and mature pollen)	Chaturvedi et al. (2013)
*Solanum lycopersicum (cv. 3017)*	Pollen developmental stages un mild heat treatment (i.e., post-meiotic and mature pollen)	Chaturvedi et al. (2015)
*Solanum lycopersicum (cv. Red Setter and cv. Moneymaker)*	Membrane	Paul et al. (2015)
*Oryza sativa*	Mature pollen and coat	Dai et al. (2006)
	Mature and germinated pollen	Dai et al. (2007)
*Lilium davidii*	Plasma membrane from mature and germinated pollen	Han et al. (2010)
*Lilium longiflorum*	Membrane, organelle from pollen tube	Pertl et al. (2009)
Maize (*Zea mays*)	Pollen coat	Petersen et al. (2006)
*Pinus strobus*	Pollen tube	Fernando (2005)
*Pinus bungeana*	Pollen tube treated with nifedipine	Wu et al. (2008)
*Picea meyeri*	Pollen tube treated with trifluoperazine and latrunculin B	Chen et al. (2006) and Chen et al. (2009)
Tobacco	Pollen developmental stages and pollen tube	Ischebeck et al. (2014)
Tobacco	Mature and pollen tube (phosphoproteomic analysis)	Fila et al. (2012, 2016)
*Parietaria judaica*	Mature pollen	Barranca et al. (2010)
*Picea wilsonii*	Mature pollen and germinated pollen	Chen et al. (2012)
*Zea mays* L.	Pollen	Cosgrove et al. (1997)

## Proteomic studies on pollen under temperature stress treatment

All studies so far have provided a vital information to understand many crucial and complex processes of pollen development. It is also clearly evident that proteomics data are important to complement transcriptomic analysis to determine pollen functionality.

Recently, plant response to heat stress has been reviewed in detail by Bokszczanin et al. (2013), but proteomic knowledge to understand the course of pollen development under harsh environmental condition (e.g., heat stress) is very limited. In contrast, organ-specific proteome analysis under heat stress condition in a variety of crop species is well reviewed (Kosova et al. 2011). Proteomic analysis of the anthers (at anthesis stage) from three different varieties of rice under high temperature determined the presences of cold- and heat-shock proteins (Jagadish et al. 2010). Giorno et al. (2010) determined the accumulation of the proteins HsfA2 and Hsp 17-CII in the young anthers and mature microspores under heat stress condition.

Studies reveal that pollen development of rice is sensitive to lower temperatures which lead to the pollen sterility and loss of yield. cDNA microarray analysis of rice anther (microspore release stage) determined around 160 transcripts showing up- and downregulation under cold stress (Yamaguchi et al. 2004). Further, proteomics studies of rice anthers (young microspore stage) showed that cold stress enhances or induces protein degradation. Protein candidates like HSP 70, β-expansin, 2, 3-bisphospho-glycerate-independent phosphoglycerate mutase and glycogen phosphorylase are degraded under cold stress condition which possibly lead to pollen sterility (Imin et al. 2004).

Recently, in a proteomic study on tomato pollen (ecotype Hazera 3017) under mild heat stress condition considering two developmental stages (i.e., post-meiotic and mature) unique protein candidates have been detected which showed significant changes in the concentrations compared to the control (Chaturvedi et al. 2015). Based on identification of proteins like LEA, HSP 20 and HSP 22, chaperone protein htpG from post-meiotic stage, it can be concluded that the majority of the proteins are synthesized to protect the normal developmental process under mild heat stress condition. Increased levels of heat-responsive proteins might hint to the processes of acquired thermotolerance which has to be investigated in future. Considering mature pollen, the majority of heat-responsive proteins are involved in energy-related processes which are essential for pollen germination and tube growth. This observation leads to the conclusion that mild heat stress condition does not impair mature pollen for undertaking the process of germination but rather leads to rapid acclimatization responses to prepare the pollen for harsher conditions. This analysis was performed by introduction of a novel feature of MAPA (mass accuracy precursor alignment; see also above) which allows the extraction and alignment of proteotypic peptide precursor ions from complex shotgun proteomics data for accurate quantification of the unique proteins. This strategy circumvents the problem of confusing the quantification of proteins due to undistinguishable protein isoforms by a typical shotgun proteomics approach.

## Developmental priming

**Fig. 3:**
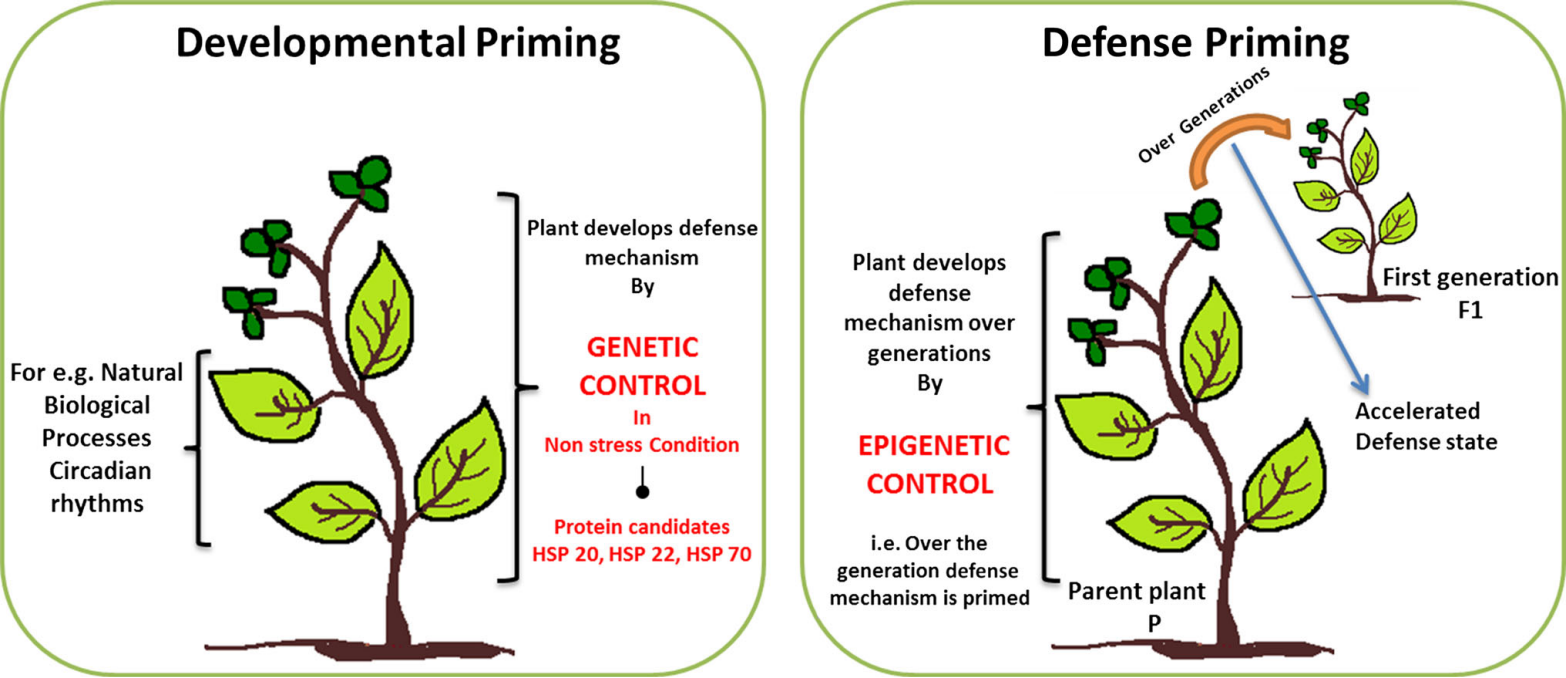
Schematic representation of developmental priming in contrast to defense priming. Developmental priming is a genetic control program which provides defense mechanism during developmental processes under non-stressed condition for, e.g., genes with defense mechanism (HSP 20, HSP 22, and HSP 70). In contrast, defense priming is linked to epigenetic control that is initiated by environmental stresses leading to defense mechanisms in the next generations

Developmental priming is a genetic program during development which controls not only genes involved in cell differentiation but also genes typically known from defense mechanism against environmental stresses and genes involved in later stages of development (Chaturvedi et al. 2013). For example, in mature pollen the rapid tube growth during germination of the dehydrated pollen might be supported by presynthesized proteins. In contrast defense priming is initiated by environmental stresses. Those defense priming processes may lead to epigenetic adaptation (Fig. 3).

Priming is an important concept in plant stress physiology to explain defense mechanisms. According to Conrath et al. (2015), priming can be defined as “sensitization of a cell or organism for enhanced defense; causes faster and more robust activation of defense response upon challenge.”

Over the past two decades there has been increasing evidence that priming plays a very important role in plants to provide a local and systemic immunity against pest, microbes, biotic and abiotic stresses. Hence, it is observed that primed plants possess enhanced defense power compared to non-primed plants. For example, cucumber plants infected by the fungus *Colletotrichum lagenarium* showed strong defense against the second penetration attempt by rapidly depositing the papillae at the point of pathogen attack (Kuc 1982). This analysis led to the conclusion that hypertensive response was induced in the plants that had previously experienced the pathogen attack (Ross 1961; Kuc 1995).

Priming is also studied in detail in animals, for example, enhanced defense mechanisms of macrophages and mammalian monocytes against bacterial lipopolysaccharides (LPS). In the recognition of LPS, macrophages and monocytes produce cytokines (TNF-α and IL-1β) that can combat the effect of LPS (Raetz et al. 1991). This primed state of macrophages and monocytes can even combat lower dose of LPS which is due to endogenous protein interferon-γ. This protein plays an important role in signaling mechanism of the mammalian cells which are attacked by the pathogen (Gifford and Lohmann-Matthes 1987; Koerner et al. 1987; Hayes et al. 1995b). It has also been suggested that priming effects for cellular defense can also be mediated at transcript level in macrophages and monocytes (Hayes et al. 1995a). Similarly, there are several studies which briefly review the process of priming in plants providing the insight on the importance of priming mechanisms (Conrath et al. 2015).

In our recent study, the generated quantitative proteome map from the tomato pollen developmental stages [i.e., microsporocyte (pollen mother cells), tetrad, microspore, polarized microspore and mature pollen] revealed a process in which the cells of a specific stage are prepared for the effective progression of development and potentially “primed” for sudden environmental stress (Chaturvedi et al. 2013). In this study, each stage showed a specific reprogramming of the proteome. Since it was observed that many proteins which are typically involved in stress responses (e.g., HSP 70, HSP 22, HSP 20) were also identified during the normal unstressed developmental process, these specific responses were termed as developmental priming (Chaturvedi et al. 2013).

The concept of developmental priming is relatively novel, and in the following, we summarize and collect some preknowledge and hypotheses.

## Genes and proteins involved in developmental priming

In the study performed by Chaturvedi et al. (2013), it was observed that heat-shock proteins like HSP 20, HSP 22 and HSP 70 showed increased levels in the early developmental stages under control condition. It is hypothesized that these proteins might protect the early stages of pollen development against sudden fluctuating temperatures. On the other hand, these proteins do play an important role in cellular processes like protein folding, protein protection and transportation. Thus, it can be speculated that these proteins maintain the functionality of the developmental process.

Heat-shock proteins (HSPs) are highly homologous proteins which are expressed in cells either constitutively or in cell cycle or in a developmental process. The role of HSPs in plants is reviewed in detail by Vierling (1991). There are studies which provide evidence that HSPs are present during the embryogenesis and pollen development (Winter and Sinibaldi 1991). Some members of the HSP family are expressed in the absence of heat stress, and they are known as cognate HSPs including HSP 70 referred as Hsc 70 (Craig 1985). Hsc 70 is involved in specific functions like clathrin and ATPase uncoating (Ungewickell 1985; Chappell et al. 1986). Munro and Pelham (1986) demonstrated that a protein related to the HSP 70, expressed in normal rat liver is synthesized under glucose starvation. Davis et al. (1986) demonstrated that transferrin receptor is related to clathrin-uncoating ATPase/heat-shock protein. Some of these Hsc proteins are expressed during the normal development of tomato (Duck et al. 1989). An experimental study performed by Duck and Folk (1994) clearly demonstrated that Hsc 70 proteins are synthesized during the early pollen development and stored in mature and germinating tomato pollen. In the case of tomato, mature and germinating pollen upon heat stress does not synthesize new HSP 70 proteins to combat stress, unlike other plant species; it rather utilizes cognate HSP 70 proteins which are stored in mature pollen. Pollen is the most sensitive tissue of the plant, and its developmental process is extremely fragile to the environmental stresses; hence, it may not have the capacity to rapidly produce new heat-shock proteins. During early pollen development the nurse cells (or tapetal cells) provide nutrition to the pollen mother cells via secretory pathways. Since the Hsc 70 proteins play an important role in intracellular transport activities, it is possible that Hsc 70 protein expressed during early pollen development might get involved with the transport activities of tapetum and get stored in mature pollen. The transport-related activities of Hsc/HSP 70 are already being demonstrated in several studies (Chirico et al. 1988; Deshaies et al. 1988; Miernyk et al. 1992) where they are involved in transportation of precursor proteins into the endoplasmic reticulum and mitochondria. A further study performed by Duck et al. (1989) also demonstrated the expression of Hsc 70 in vegetative and reproductive organ of *Lycopersicon esculentum*; in this analysis, tomato tissues showed high expression of Hsc 70 transcripts including secretory tissues or organs with rapidly dividing cells (e.g., developing tapetum of immature anther, inner envelope of developing seed and all tissues that prepare glycoprotein and secretory protein). But in this analysis no evidence was provided for the expression of Hsc 70 gene in mature pollen.

The findings by Duck et al. (1989) were very important, and they showed a clear correlation with the recent shotgun proteomics analysis performed by Chaturvedi et al. (2013). It also strongly provided the support to the concept of developmental priming. It is evident that some genes/proteins might get presynthesized over the process of pollen development and stored in the dormant state. Since pollen development is a cell-specific sequential process, upon sudden stress condition these proteins are activated and provide a first line of defense at the molecular level. The interplay of translational or transcriptional activity during these processes has yet to be confirmed in tomato tissues. *Xenopus oocytes* stores HSP 70 transcripts which are later translated specifically in the stress condition (Bienz and Gurdon 1982). Frova et al. (1989) reported that HSP 72 and 64 kDa are synthesized during pollen development of maize (Stage C: trinucleate pollen maturation) at 25 and 38 °C, and this study was performed using 1D SDS-PAGE. Expression of HSP 18 KDa during microsporogenesis and gametophyte development of maize was also reported by Atkinson et al. (1993). Germinating pollen of lily, *Petunia* and *Tradescantia* also showed similar results (Xiao and Mascarenhas 1985; Schrauwen et al. 1986). However, regulation of the stored cognate HSP proteins in pollen of other plant species has yet to be explored in detail. A study reported by Prandl and Schoffl (1996) determined the role of heat-shock element (HSE) sequences and heat-shock transcription factor in developmentally regulated expression.

Several other studies also determined the role of cognate HSPs in various plant tissues; for example, heat-shock cognate protein 80 (Hsc 80) showed expression in shoot and root apical of tomato (*Lycopersicon esculentum*) during normal development (Koning et al. 1992). A study performed by Palter et al. (1986) demonstrated the expression of Hsc 70 in *Drosophila* tissues, where the highest expression was observed in ovaries and embryos compared to larva, pupae and adults. Joanisse et al. (1998) reported a brief review on small heat-shock proteins (sHsps) and their cell-specific expression in the developmental tissues of *Drosophila melanogaster* (DM) under normal condition (i.e., in the absence of stress), and some sHsps like HSP 23, HSP 26 and HSP 27 showed expressions in male gametogenesis of *DM* i. The expression of HSP 23 was restricted to cells of somatic lineage, HSP 26 expression levels were high in early embryos, and HSP 27 was mainly expressed in the germ line cells and somatic cells. Under heat-shock condition, HSP 22 and HSP 70 were induced via heat-shock transcription factor (HSF).

## Conclusion

In recent years, advancement in the functional genomics studies has widely opened the areas of scientific research which also include OMIC analysis. Large levels of transcriptomic work have been performed in different plant tissues and cells, although transcriptional level information does not reflect or identify molecular processes at proteome or metabolome levels. As a consequence, proteomic analyses are a major milestone to support our understanding at cellular and molecular level of the cell/tissue type.

Proteomics of highly reduced male gametophyte revealed processes of cell wall remodeling, alterations in metabolism, protein fate, signaling and reservoirs of carbohydrate and energy metabolism.

Recently, cell-specific reference proteomes of tomato and tobacco pollen development were generated which included microsporocytes (pollen mother cells), tetrads, microspores, polarized microspores, mature pollen and pollen tube growth (Chaturvedi et al. 2013; Ischebeck et al. 2014). Each stage showed a specific reprogramming of the proteome. These specific responses in pollen development process were termed “developmental priming” as opposed to “defense priming” (Chaturvedi et al. 2013). Here is the hypothesis that a genetic or epigenetic program controls the presence of protective proteins such as heat-shock proteins already in the non-stressed state, to compensate for sudden changes in temperature during the maturation of the pollen. Further studies, especially the integration of transcriptomics and proteomics during development and under temperature treatment, are necessary to validate these hypotheses. Due to rapid development in analytical technologies, there is a huge amount of proteome and metabolome data available. One of the greater challenges is to validate, understand and integrate the large data which can provide systemic knowledge about the molecular function and networks of a cell/tissue. Future research on pollen requires refinement of experimental designs and use of appropriate technology to allow for better understanding of this fascinating and complex process. In particular, the impact of identified protein candidates involved in developmental priming will be tested with respect to their effect on pollen viability under fluctuating environmental conditions. Based on the better understanding of protein dynamics involved in pollen development and stress protection, smart breeding strategies can be designed for more resistant plants and higher productivity in harsh environments (Weckwerth 2011).

### **Author contribution statement**

 P.C., A.G. and W.W. designed the study, analyzed the data and wrote the manuscript.
